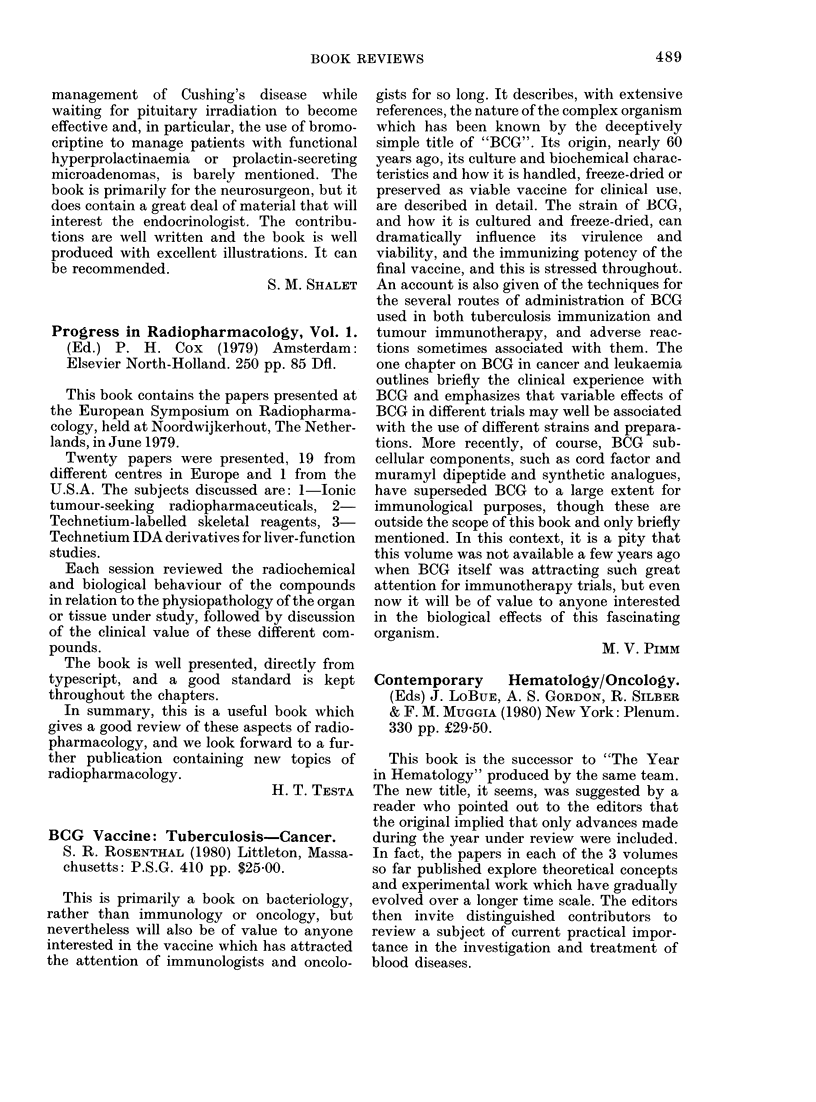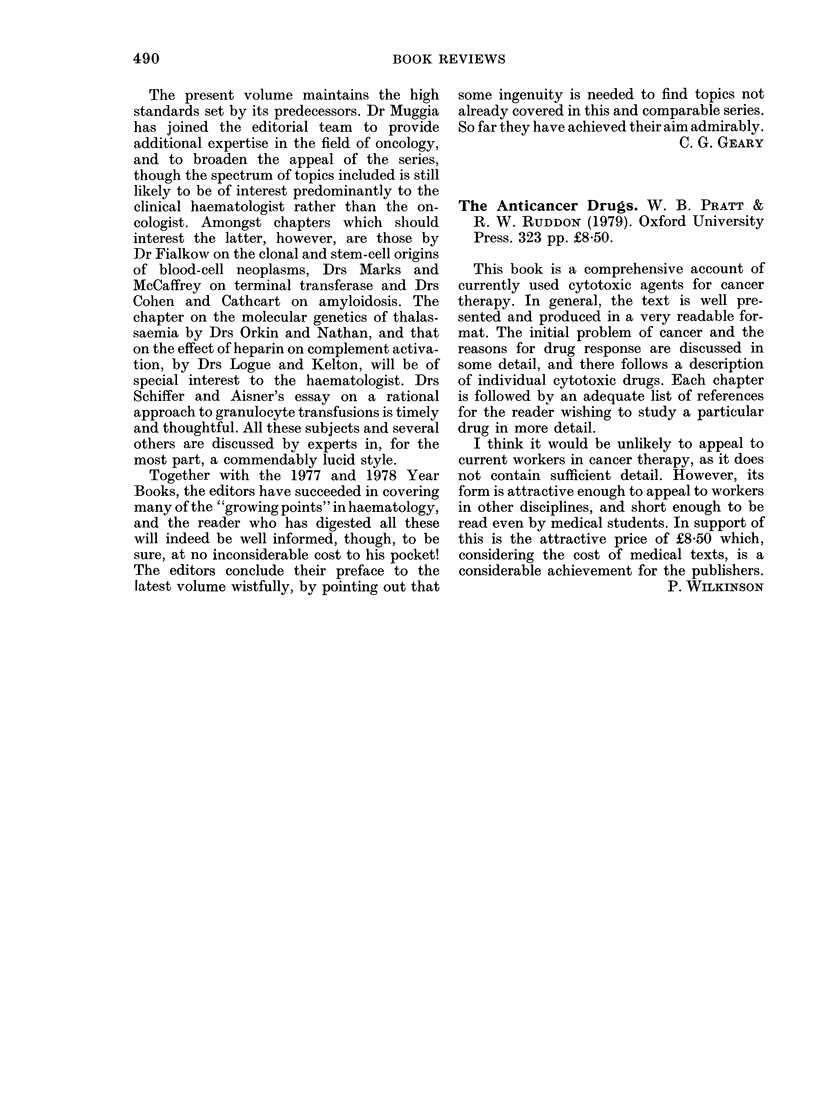# Contemporary Hematology/Oncology

**Published:** 1980-09

**Authors:** C. G. Geary


**Contemporary Hematology/Oncology.** (Eds) J. LOBUE, A. S. GORDON, R. SILBER & F. M. MUGGIA (1980) New York: Plenum. 330 pp. £29·50.

This book is the successor to "The Year in Hematology" produced by the same team. The new title, it seems, was suggested by a reader who pointed out to the editors that the original implied that only advances made during the year under review were included. In fact, the papers in each of the 3 volumes so far published explore theoretical concepts and experimental work which have gradually evolved over a longer time scale. The editors then invite distinguished contributors to review a subject of current practical importance in the investigation and treatment of blood diseases.

The present volume maintains the high standards set by its predecessors. Dr Muggia has joined the editorial team to provide additional expertise in the field of oncology, and to broaden the appeal of the series, though the spectrum of topics included is still likely to be of interest predominantly to the clinical haematologist rather than the oncologist. Amongst chapters which should interest the latter, however, are those by Dr Fialkow on the clonal and stem-cell origins of blood-cell neoplasms, Drs Marks and McCaffrey on terminal transferase and Drs Cohen and Cathcart on amyloidosis. The chapter on the molecular genetics of thalassaemia by Drs Orkin and Nathan, and that on the effect of heparin on complement activation, by Drs Logue and Kelton, will be of special interest to the haematologist. Drs Schiffer and Aisner's essay on a rational approach to granulocyte transfusions is timely and thoughtful. All these subjects and several others are discussed by experts in, for the most part, a commendably lucid style.

Together with the 1977 and 1978 Year Books, the editors have succeeded in covering many of the "growing points" in haematology, and the reader who has digested all these will indeed be well informed, though, to be sure, at no inconsiderable cost to his pocket! The editors conclude their preface to the latest volume wistfully, by pointing out that some ingenuity is needed to find topics not already covered in this and comparable series. So far they have achieved their aim admirably.

C. G. Geary